# Association of Reported Collaboration Quality with Hospice Providers and Hospice Utilization in Assisted Living

**DOI:** 10.1093/geroni/igaf122.704

**Published:** 2025-12-31

**Authors:** Xiao (Joyce) Wang, Joan Teno, Melissa Clark, Kali Thomas, Pedro Gozalo, Emmanuelle Belanger

**Affiliations:** Brown University, Providence, Rhode Island, United States; Brown University, Providence, Rhode Island, United States; Brown University, Providence, Rhode Island, United States; Johns Hopkins University, Baltimore, Maryland, United States; Brown University, Providence, Rhode Island, United States; Brown University, Providence, Rhode Island, United States

## Abstract

Hospice is crucial in meeting the increasing care needs of dying residents in assisted living (AL) and supporting dying in place. We know little about how AL-hospice collaboration relates to staying in AL at the end of life. We conducted a national survey of AL administrators in 2021-2023 about end-of-life care processes in large ALs (25+ beds). The survey contained instruments adapted from other long-term care settings, including a Likert-scale with items about AL-hospice collaboration quality (e.g., AL staff know when to expect a hospice nurse visit for all/most/some/none of the time). The sample included 9,069 AL residents who died in 2021 or 2022 in 1,501 surveyed ALs. We operationalized “staying in place with support at the end of life” as the number of days with hospice in AL in the last 90 days of life, excluding days receiving hospice in other settings. Two-part models were used to estimate the relationship between reported AL-hospice collaboration quality and days receiving hospice in AL, accounting for individual-, AL-, and market-level characteristics. Decedents spent 2.6 more days on hospice in AL (95% CI: 1.1, 4.2) if administrators reported residents were always assessed within 24 hours of hospice referrals, compared to residents in ALs where this happened none/some/most of the time. This difference represents 11% more days in place for residents who lived in ALs where administrators reported that hospice providers always respond timely to hospice referrals. The collaboration between hospice and AL staff is a modifiable factor that can improve end-of-life care experiences.

